# FerA is a Membrane-Associating Four-Helix Bundle Domain in the Ferlin Family of Membrane-Fusion Proteins

**DOI:** 10.1038/s41598-018-29184-1

**Published:** 2018-07-19

**Authors:** Faraz M. Harsini, Sukanya Chebrolu, Kerry L. Fuson, Mark A. White, Anne M. Rice, R. Bryan Sutton

**Affiliations:** 1 Department of Cell Physiology and Molecular Biophysics, Texas Tech University Health Sciences Center, Lubbock, TX 79430-6551 USA; 2 Department of Biochemistry and Molecular Biology, The University of Texas Medical Branch, Galveston, TX 77555 USA; 3 Department of Biophysics, Johns Hopkins University, Baltimore, MD 21205 USA; 4 Center for Membrane Protein Research, Texas Tech University Health Sciences Center, Lubbock, TX 79430-6551 USA

## Abstract

Ferlin proteins participate in such diverse biological events as vesicle fusion in *C. elegans*, fusion of myoblast membranes to form myotubes, Ca^2+^-sensing during exocytosis in the hair cells of the inner ear, and Ca^2+^-dependent membrane repair in skeletal muscle cells. Ferlins are Ca^2+^-dependent, phospholipid-binding, multi-C2 domain-containing proteins with a single transmembrane helix that spans a vesicle membrane. The overall domain composition of the ferlins resembles the proteins involved in exocytosis; therefore, it is thought that they participate in membrane fusion at some level. But if ferlins do fuse membranes, then they are distinct from other known fusion proteins. Here we show that the central FerA domain from dysferlin, myoferlin, and otoferlin is a novel four-helix bundle fold with its own Ca^2+^-dependent phospholipid-binding activity. Small-angle X-ray scattering (SAXS), spectroscopic, and thermodynamic analysis of the dysferlin, myoferlin, and otoferlin FerA domains, in addition to clinically-defined dysferlin FerA mutations, suggests that the FerA domain interacts with the membrane and that this interaction is enhanced by the presence of Ca^2+^.

## Introduction

Ferlins are a relatively new class of large, multi-domain, type II transmembrane proteins that have been implicated in a wide variety of biological functions centered on membrane fusion events. The founding member of the ferlin family, fer-1, was first described in *C. elegans* as a protein that was required for Ca^2+^-mediated fusion of membranous organelles with the sperm’s plasma membrane during spermatogenesis^1,2^. Later a homologous gene, known as *misfire*, was identified in *Drosophila* and implicated in sperm activation, egg patterning, and plasma-membrane breakdown in germ cells^3^. Since the discovery of these genes in *C. elegans* and *Drosophila*, six paralogs have been identified in mammalian genomes and have been annotated as FER1L1 - FER1L6^4^. All six ferlins are composed of multiple tandem C2 domains, a centrally-positioned FerA domain, and a single C-terminal transmembrane helix. Most, but not all ferlins possess a DysF domain. X-ray and NMR structures of the C2A domains of dysferlin^5^, myoferlin^6^, and otoferlin^7^, as well as the DysF domains of dysferlin^8^ and myoferlin^9^ are currently known.

Dysferlin (Fer1L1), myoferlin (Fer1L3), and otoferlin (Fer1L2) participate in a form of membrane fusion that is similar in many ways to Ca^2+^-dependent exocytosis. In neuronal exocytosis, v-SNAREs found on synaptic-vesicle membranes form a stable four-helix bundle with complementary t-SNAREs on the target membrane at the active zone of neurons^10^. The SNARE bundle then interacts with the C2 domain-containing protein, synaptotagmin, in a Ca^2+^-dependent manner to trigger the fusion of vesicle and target membranes. Similarly in membrane repair, patching vesicle proteins form a complex with proteins that localize to damaged target membranes and mediate the fusion of patching vesicles to the site of damage; Ca^2+^ triggers this fusion event as it does in neuronal exocytosis^5,11,12^. The mechanism that describes this process is known as patch repair^13,14^. Briefly, damage to cell membranes stimulates the recruitment of proteins such as MG53^15^, annexinA6^16^, and dysferlin to an annulus of negatively-charged phospholipids that focus around lesions in the sarcolemma^14^. MG53 and annexinA6 are soluble proteins that localize to the target membrane whereas dysferlin is tethered to a patching vesicle via a single C-terminal transmembrane helix. The recruitment and fusion of these patching vesicles by dysferlin to the site of injury occur rapidly, usually within one second^14^. A lack of dysferlin in muscle cells causes an accumulation of unfused vesicles near sites of injury^13^. Inadequate amounts of dysferlin protein^17^ and errant dysferlin function due to mutations^18^ have been linked to Limb-Girdle Muscular Dystrophy-Type 2B in humans.

Myoferlin and otoferlin are two additional ferlin family members that have received considerable attention. Myoferlin is expressed in myoblasts and shares a considerable amount of sequence similarity with dysferlin, yet their physiological roles are different^19^. Myoferlin is present earlier in development as it fuses myoblasts to form myotubes in the formation of skeletal muscle^20^. Otoferlin is expressed exclusively in the hair cells of the inner ear, and it may substitute for some of the Ca^2+^-dependent triggering activities of synaptotagmin^21,22^; however, the absolute function of otoferlin is still under debate^23^. Therefore, from a physiological perspective, ferlin proteins serve an essential role in membrane fusion. What is not yet understood is whether ferlins possess the fusion machinery to join disparate phospholipid membranes by themselves, or whether they require other partners as a part of a larger multi-protein fusion complex.

We have established by secondary structure analysis, circular dichroism (CD), and small-angle X-ray scattering (SAXS) that the FerA domain is *α*-helical and globular in solution. We have also constructed 3D models of FerA, and we conclude that it is a new four-helix bundle fold. However, unlike other four-helix bundles, FerA domains are unique in that they can bind to, and perhaps insert into, phospholipid membranes in a Ca^2+^-dependent manner. Therefore, the ferlin fusion mechanism may share similar biophysical characteristics to both eukaryotic SNARE-based proteins and viral fusion proteins.

## Results

### Primary and Secondary Structure Assessment of the Ferlin FerA Domain

**Figure 1:**
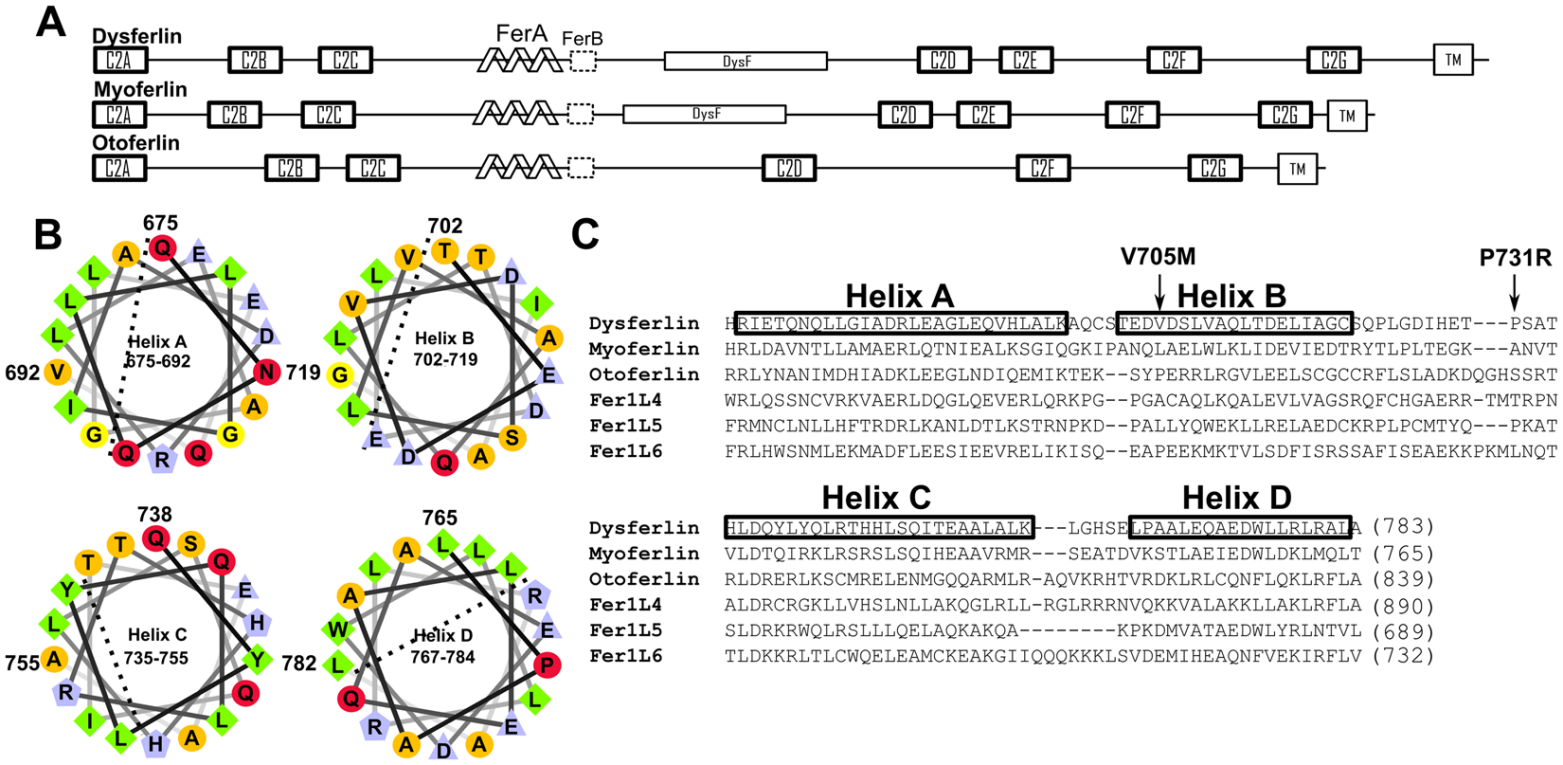
(**A**) Schematic structures of dysferlin, myoferlin, and otoferlin. The linkers between each folded domain are drawn to scale. Bolded boxes denote C2 domains, schematic helices reflect the FerA domain, dotted boxes are the FerB domains. (**B**) Helical wheel representation of a portion of the helices in human dysferlin FerA to highlight their amphipathic nature. The dashed lines demarcate hydrophobic-hydrophilic boundaries^56^. Residue numbers for human dysferlin FerA are provided for the amino- and carboxyl- terminal residues of each helix. (**C**) PROMALS (PROfile Multiple Alignment with predicted Local Structures) alignment of the FerA domain from human dysferlin, myoferlin, and otoferlin, Fer1L4, Fer1L5, and Fer1L6. Boxes demarcate the *α*-helices labeled A-D. The residue ranges provided are from the human dysferlin sequence. Residue numbers for the carboxyl- terminal residues of each FerA domain are provided in parentheses. Arrows pointing to V705M and P731R denote the positions of the two LGMD-2B FerA mutations in dysferlin described herein.

The FerA domains are located near the center of the ferlin proteins primary sequence. The domain is typically surrounded by a set of three C2 domains on its N-terminal side and a DysF domain followed by at least three C2 domains on its C-terminal side. Otoferlin is the exception, as it does not have a DysF domain. Secondary-structure prediction of the human dysferlin protein using SMART database tools shows a 66 amino acid FerA consensus sequence composed of two *α*-helical segments^24^. However, when including additional primary sequence before and after the SMART definition, two additional consensus *α*-helices can be predicted in all ferlin proteins in the family. Altogether the boundaries of the complete four-helix FerA domain are capped by disordered consensus residues between C2C on the amino-terminal side and the predicted FerB sequences on its C-terminal side (Fig. 1). A more inclusive description for FerA is, therefore, four long amphipathic *α*-helices, where two groups of two *α*-helices are separated by a long, central connecting linker (Fig. 1C). We have labeled the helices as A, B, C, and D. The loops between helices A-B and C-D are less than 4 residues in length, while the linker between B and C is less than 17 residues in length (Fig. 1B,C).

The technique used to purify dysferlin, myoferlin, and otoferlin FerA confirms that each domain is unique with respect to charge (Table S1). Each was purified as bacterially expressed, soluble fusion proteins (purification methods are detailed in the Supplemental Information) (Fig. S1). The FerA components were enzymatically cleaved to separate the FerA domains from their His-tagged maltose binding protein (MBP) fusion partners. We continued the purification with IEC (ion-exchange chromatography). Interestingly, dysferlin FerA bound to a QAE-Sepharose column, suggesting that its overall charge at physiological pH is predominately negative. Under identical expression and purification conditions, the overall charge of myoferlin FerA was close to neutral as it did not bind to either SP-Sepharose or QAE-Sepharose under any condition that we tried. Otoferlin FerA, on the other hand, bound to SP-Sepharose; therefore, it is predominately positively charged at physiological pH.

As some of the ferlins currently have no known functions, their FerA domains can be clustered by charge with other FerA domains to provide some insight into their functions. In a comparison of all six ferlin proteins (Fig. 1C, Table S1), Fer1L5 and Fer1L6 cluster with otoferlin and myoferlin, respectively. For example, the FerA domains of otoferlin (+8.5) and Fer1L5 (+9.1) are similar with respect to charge. Both myoferlin (−1.6) and Fer1L6 (−0.6) share near-neutral net charges. Dysferlin and Fer1L4 are the outliers. Dysferlin has a strong negative net charge at −8.4, while Fer1L4 is intensely positively-charged at +20.3. Such charge diversity in one domain within a protein family can indicate electrostatic interactions used for specific biological functions. These functions include specific protein-protein interactions^25^, Ca^2+^-phospholipid bridging as a link between the protein domain and the phospholipid membrane^26^, or perhaps protein interactions with negatively-charged phospholipids^27^.

**Figure 2:**
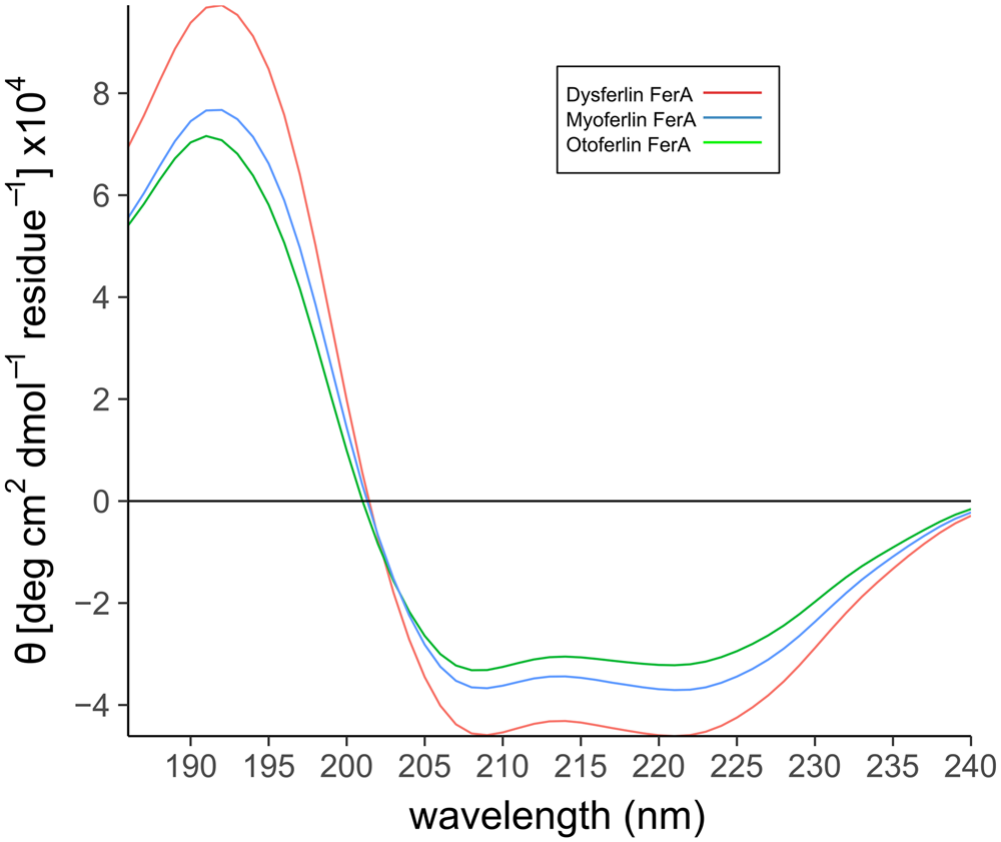
Circular Dichroism (CD) spectra of isolated FerA domains, demonstrating the secondary structure of various FerA domains. All spectra are consistent with the CD signature of *α*-helical proteins. Dysferlin FerA (red), myoferlin FerA (blue), otoferlin FerA (green).

The secondary structure of a soluble protein can be estimated by correlating the CD spectrum of an unknown sample against a library of CD spectra of known secondary structures^28,29^. In the case of the FerA domain, we used CD analysis to confirm that our purified proteins were folded and that each of the ferlin FerA domains was similar with respect to their secondary structure. Curve fitting of the CD spectrum of wild-type human dysferlin FerA corresponds to 94% *α*-helix (Table S3). Two mutations have been defined within the FerA domain of dysferlin that have been linked to either Miyoshi Myopathy (V705M) or Limb-Girdle Muscular dystrophy (P731R). These two mutations in two dysferlin FerA mutants have virtually the same fraction of *α*-helix as wild-type FerA (Table S3); therefore, neither V705M nor P731R interferes with the overall secondary structure of the dysferlin FerA domain. Myoferlin FerA has 86% *α*-helix whereas otoferlin has 95% *α*-helix (Table S3) (Fig. 2).

### Molecular Models of FerA

Crystallization trials of dysferlin, myoferlin, and otoferlin FerA domains failed to produce crystals for analysis. Further, our initial NMR analysis of dysferlin FerA yielded promising HSQC spectra; however, inter-helical motion of the FerA domain while collecting the 3D data made structure determination less tractable (data not shown). As there are no 3D structures predicted to be homologous to FerA in the PDB (Protein Data Bank), we computed a series of molecular models using Modeller^30^ and Robetta^31^ to obtain an unbiased set of *de novo* models of the FerA domain with which to interpret subsequent SAXS data. The PDB entry listed as ‘2QUP’ served as a suitable four-helix bundle template^32^. 2QUP is an uncharacterized four-helix bundle protein isolated from *Bacillus halodurans* and is about 25% similar to the dysferlin FerA primary sequence (Fig. S4). The relatively high similarity between the FerA sequence and the 2QUP sequence is due to the relatively high surface charge and compact size of the two bundles, and not necessarily on any evolutionary relatedness between dysferlin FerA and 2QUP. The dysferlin FerA primary sequence was also exported to Robetta for *de novo* independent modeling. Robetta returned several four-helix bundle models that we used in subsequent analyses.

**Figure 3:**
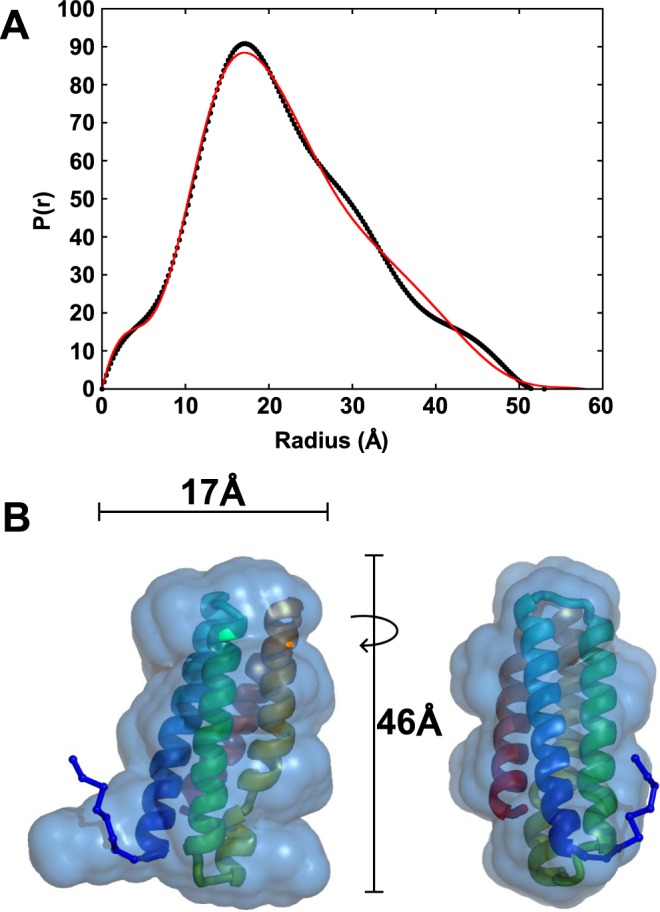
(**A**) The pair-distance distribution function, P(r) for the dysferlin FerA four-helix bundle. The P(r) calculated from GNOM (∙) and the best-fit CORAL model’s theoretical P(r) (red-line) are shown for comparison. (**B**) SAXS-constrained FerA *ab initio* and rigid-body models. The superpositioned GASBOR *ab initio* bead model and a Robetta based CORAL model in perpendicular side views. The CORAL model’s missing N-terminal residues were modeled as a flexible C *α* chain. The polypeptide chain progresses along a rainbow of color from a blue N-terminus to a red C-terminus. The additional N-terminal residues from the cloning vector are included.

To determine if the isolated FerA domain was consistent with a compact globular shape, we performed SAXS analysis on the purified FerA domain. A dilution series of the FerA protein showed no concentration dependence. However, the 1.2 mg/mL data had a poor Guinier region and significant buffer subtraction problems, probably due to a poor signal-to-noise ratio. To avoid radiation-induced aggregation, only the first 1–2 frames were averaged in SAXLab to produce separate sample curves of 60 minutes total exposure (Table S4). Therefore, the FerA data used for analysis was merged from its 5 and 2.5 mg/mL curves to improve the signal-to-noise ratio. The merged FerA curve has an R_g_ (15.4 Å) and Dmax (55 Å) (Fig. 3A). A DAMMIF *ab initio* molecular shape model for FerA is a qualitatively good match to the Modeller homology model, based on the four-helix bundle PDB 2QUP, and also to most of the Robetta models (Figs 3B, S6). The higher-resolution GASBOR *ab initio* molecular shape model fits the core four-helix bundle of the top scoring Robetta models well. The homology models are missing the disordered N-terminal residues from the expression vector, which contributes to the lack of fit at larger radii. Quantitatively, the SAXS scattering curve for FerA is a good fit to the core Modeller homology model (*χ*^2^ = 1.5) and Robetta *ab initio* structures (*χ*^2^ = 1.2–1.4). Modeling the eight missing residues of the five Robetta models slightly improved the fits with a best fit of (*χ*^2^ = 0.87) and one model being rejected (Table S4). The difference between the best fitting models is minimal at this resolution (Fig. S7), and the N-terminal residues seem to be disordered, further reducing the ability for SAXS to differentiate between models.

1 $${\rm{\Delta }}{G}_{{\rm{U}}}(T)={\rm{\Delta }}{H}_{{\rm{m}}}[({T}_{{\rm{m}}}-T)/{T}_{{\rm{m}}}]-{\rm{\Delta }}{C}_{{\rm{P}}}[{T}_{{\rm{m}}}-T\mathrm{(1}-ln(T/{T}_{{\rm{m}}}))]$$

### Thermodynamic Analysis of Dysferlin FerA and Disease-Linked Mutants

**Figure 4:**
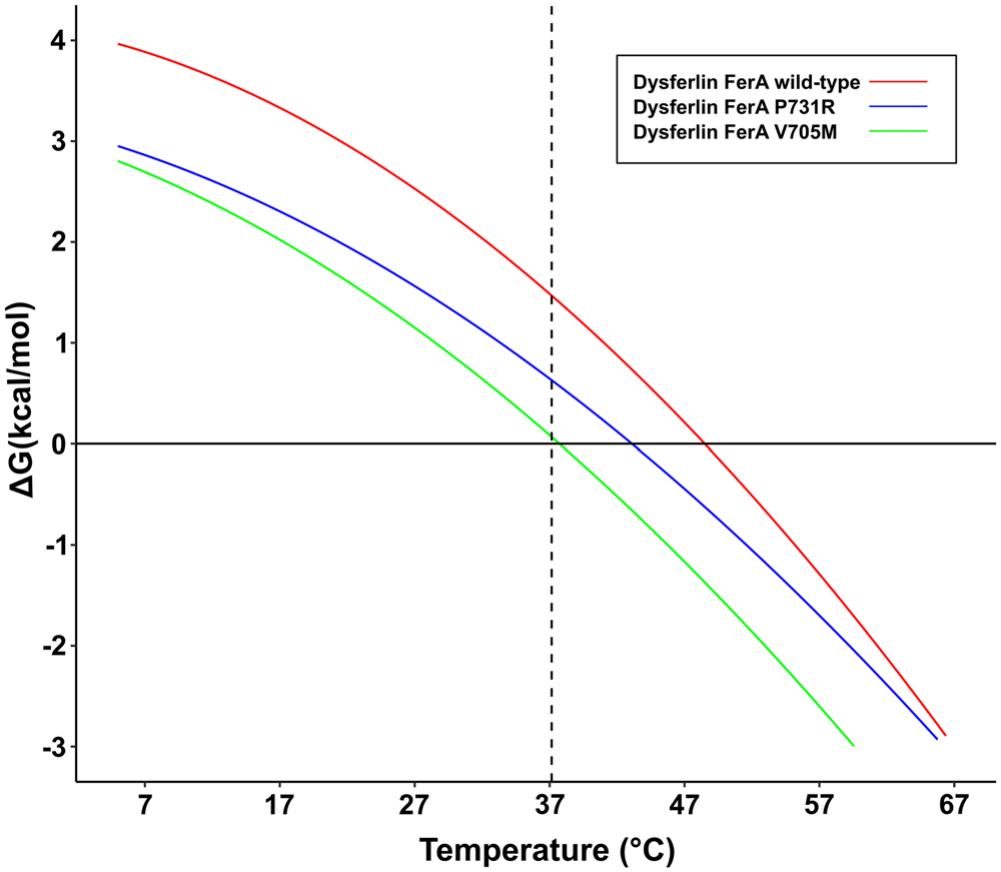
Thermal Stability Profile (ΔG versus Temperature). Free Energy Diagram of wild-type human dysferlin FerA (red), V705M (green) and P731R (blue). By definition, at 0 kcal/mol line, an equal population of native and denatured states exists. The dashed vertical line denotes 37 °C.

We studied the thermal unfolding of dysferlin FerA and the two disease-linked mutations by differential scanning calorimetry (DSC) (Table 1) and circular dichroism (CD) (Table S2, Fig. S3). All three dysferlin FerA domains showed evidence of reversible refolding using both the CD (Table S2) and DSC (Fig. S5) thermal unfolding experiments. The Gibbs-Helmholtz equation [1] was fitted to the DSC data for wild-type dysferlin, V705M, and P731R to simulate how the free energy of FerA would change over a wide temperature range (Fig. 4). We calculated a relatively low stability for wild-type dysferlin FerA (ΔG = 1.5 kcal/mol) at 37 °C. The measured change in heat capacity upon unfolding (ΔC_P_ = 0.72 kcal/mol-K) was comparable to other four-helix bundles proteins, implying a similar degree of exposure of the hydrophobic core (Table 1)^33^. Mutation of the wild-type valine residue to methionine (V705M) results in substantial destabilization of the FerA domain relative to the wild-type FerA (Table 1). Since this is a hydrophobic residue on a helix, it is likely to be a component of the hydrophobic core of the FerA four-helix bundle. In fact we measured the ΔG of unfolding for V705M to be 0.070 kcal/mol. Destabilization of V705M is likely the result of the large change in enthalpy of the domain (ΔΔH_U_ = −10.53 kcal/mol) (Table 1). One interpretation would be a reorganization of the four-helix bundle to accommodate the bulk of the mutation, which would cause a disruption or distortion of the hydrogen-bonding structure of the bundle. Indeed, the initial DSC thermogram for V705M (Fig. S5, green curve) shows indications of a modified unfolding trajectory relative to that of either the wild-type or the P731R mutant. Refolding of the V705M showed signs of domain re-annealing behavior as the thermogram appears more similar to that of wild-type FerA or P731R (Fig. S5). The other clinically-described mutation, P731R, occurs in the long linker that connects helices B and C. We measured the ΔG of unfolding for P731R to be 0.630 kcal/mol at 37 °C. This intermediate value of ΔG is consistent with the model that Pro-731 occurs in a structural linker rather than a more energetically sensitive part of the domain; however, a mutation at this locus still inflicts a large enthalpic penalty. A native-like 3D structure of the two mutants could be unfavored due to the backbone distortions required to accommodate the mutations. However, the folding of both V705M and P731R becomes entropically more favorable probably due to increased conformational flexibility in the folded state. In the case of V705M, the increased entropic contribution barely compensates for the enthalpic penalty to stabilize the domain (ΔΔH = −10.53 kcal/mol). In the case of P731R, there is a smaller enthalpic cost to the mutation (ΔΔH = −5.94 kcal/mol), and there is a smaller compensating entropic gain (ΔΔS = −5.04 kcal/mol).

**Table 1:** Calorimetric summary of thermodynamic values for the dysferlin FerA domain and clinically-derived mutations.

Domain	T_M_ (°C)	ΔC_p_ (kcal/mol K)	ΔH_cal_ (kcal/mol)	TΔS(kcal/mol)	ΔG^37 °C^ (kcal/mol)
Dysferlin FerA WT	48.35 ± 0.673	0.72 ± 0.031	42.92 ± 1.58	41.28 ± 1.58	1.470 ± 0.11
Dysferlin FerA V705M	37.58 ± 0.526	0.58 ± 0.107	32.39 ± 0.45	31.76 ± 0.83	0.070 ± 0.061
Dysferlin FerA P731R	42.95 ± 0.702	0.53 ± 0.061	36.98 ± 0.62	36.24 ± 0.61	0.630 ± 0.071

T_M_ is the value obtained from the fit of the DSC data. ΔC_P_ is the change in heat capacity. ΔH_cal_ is calorimetric enthalpy calculated by DSC. TΔS was calculated at 37 °C. ΔG was calculated by extrapolation from the Gibbs-Helmholtz equation at 37 °C. Similar T_M_, ΔH, TΔS, and ΔG values were obtained by analyzing thermal unfolding transitions from circular dichroism spectroscopy (Table S2).

**Figure 5:**
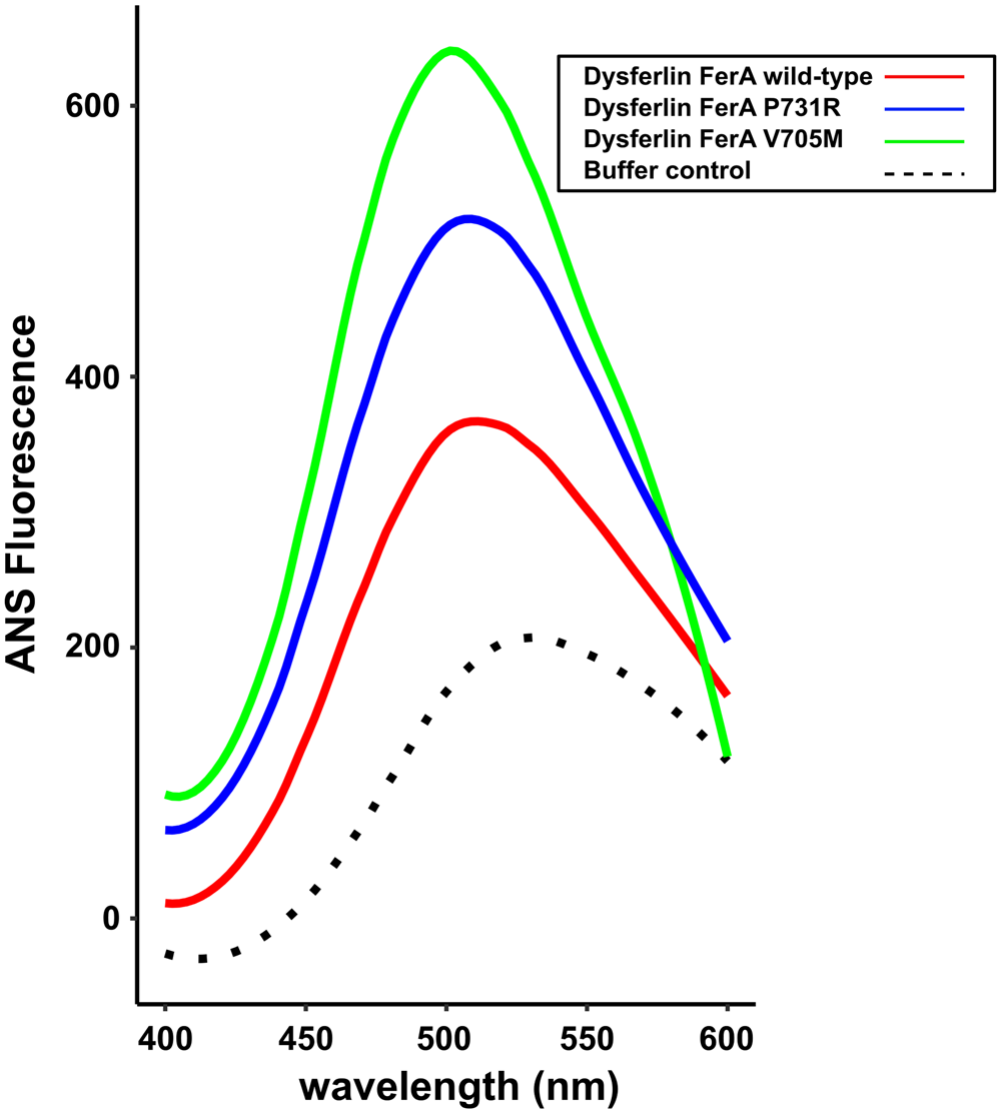
ANS fluorescence of dysferlin FerA wild-type (red), V705M (green), P731R (blue). The dotted line represents the ANS-buffer control. ANS fluorescence is reported in arbitrary units.

To test our thermal unfolding results, we used ANS (8-anilino-1-naphthalenesulfonic acid) to detect changes in the hydrophobic exposure of the dysferlin FerA domain as a function of mutation^34^. The fluorescence of ANS varies depending on its local environment. ANS also has a relatively low fluorescence in aqueous solution, and a much higher fluorescence in hydrophobic environments. Consequently, ANS has been used as an indicator for protein folding mutations and molten globule states where the hydrophobic core of a protein may be exposed to solvent^33^. From the thermal-stability profile (Fig. 4), we predict that the wild-type FerA domain should be mostly protected from ANS binding, as it should be a well packed four-helix bundle. Since the P731R has intermediate stability, we predict that it should have moderate exposure to ANS binding. By that same metric, V705M is predicted to be the least stable, and therefore, its core should have the most accessibility to ANS. Indeed, we found that wild-type FerA has a minimum accessibility to ANS dye, P731R has an intermediate value, and V705M has the most exposure. The progressive shift of the ANS fluorescence maxima toward bluer, shorter wavelengths is a further indication of a more accessible hydrophobic environment^35^ (Fig. 5). From the CD results (Fig. 2), the secondary structure is not altered by the mutations. Therefore, the four helices must be able to explore multiple helical ensembles, yet still remain soluble.

### Membrane-Binding Activity of the FerA domain

**Figure 6:**
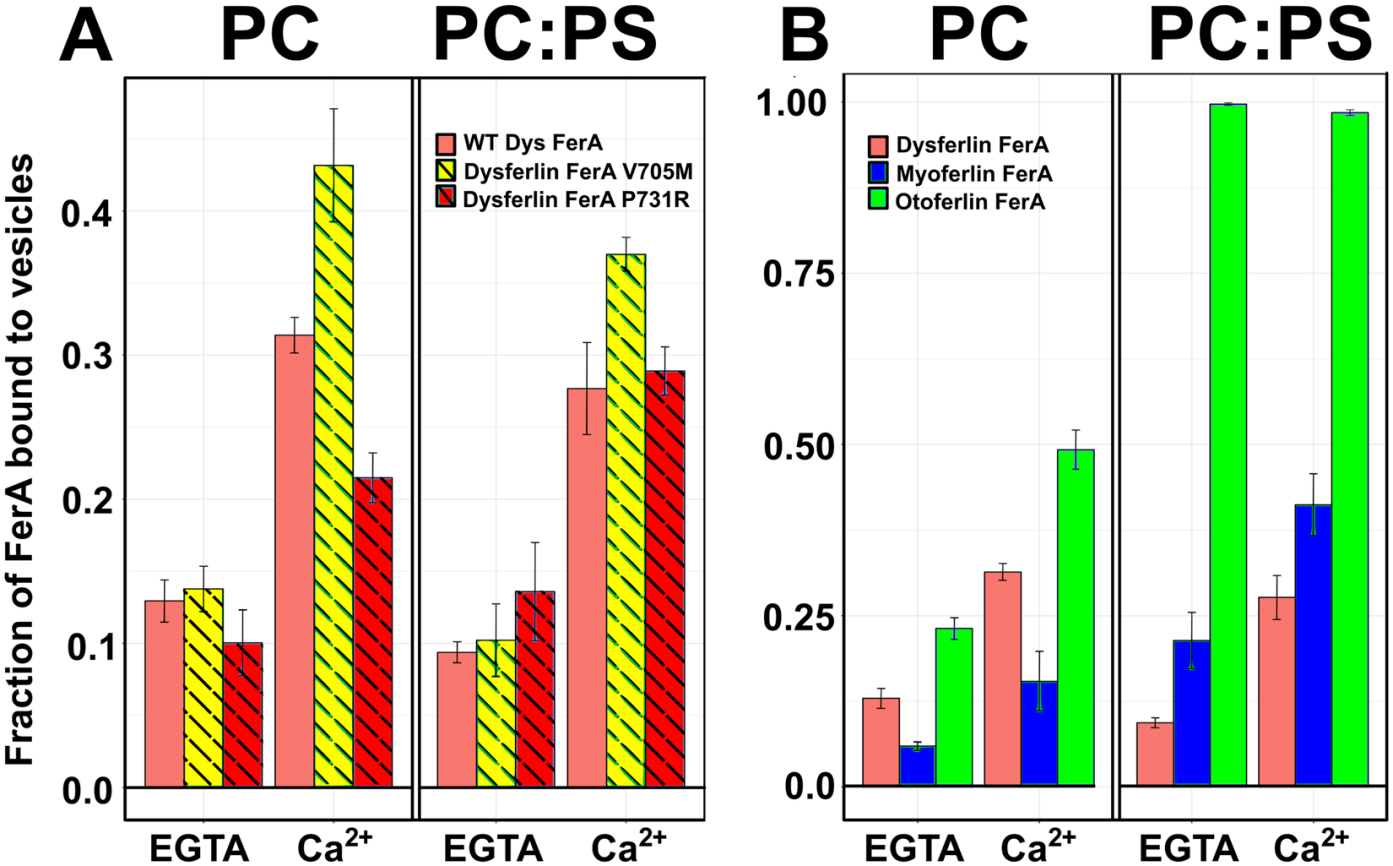
(**A**) Co-sedimentation of dysferlin FerA (solid salmon), V705M (yellow-hashed), and P731R (red-hashed). (**B**) Co-sedimentation of dysferlin FerA (solid salmon), myoferlin FerA (solid blue), and otoferlin FerA (solid green). Error bars are reported as standard deviation.

Ferlins have been implicated as membrane fusion proteins, yet there are no reports of controlled experiments showing membrane fusion activity using only ferlin proteins and membranes. Johnson and Chapman have shown that isolated otoferlin C2 domains cannot stimulate membrane fusion in the absence of SNAREs;^36^ therefore, it is possible that the FerA domain could contribute to the membrane fusion activity of the ferlin proteins. If FerA plays an integral role in the fusion activity of the ferlin proteins, one would expect similar phospholipid specificity for phospholipids as in the surrounding C2 domains^37,38^. To test whether the FerA domain binds phospholipid membranes, we conducted co-sedimentation experiments using dysferlin FerA, dysferlin mutants, Ca^2+^, and lipid vesicles of defined composition (Figs 6A, S2). Without Ca^2+^, there is minimal binding of FerA to any phospholipid mixture. In the presence of Ca^2+^ and either PC or PC:PS vesicles, there is a marked increase in binding for dysferlin FerA. Membrane binding in these FerA domains is not specific for negatively-charged phospholipid, as they bind 100% PC as well as 60% PC:40% PS liposomes. The Ca^2+^-dependent liposome binding of dysferlin FerA is accentuated by the V705M mutation, whereas the 100% PC binding of the P731R mutation is decreased. The sensitivities of wild-type FerA and P731R to negatively-charged phospholipids were similar.

In the case of myoferlin, myoferlin FerA shows increased Ca^2+^-dependent binding to negatively-charged phospholipid-containing liposomes relative to dysferlin FerA (Fig. 6B). Myoferlin FerA binds PC-only vesicles either with or without Ca^2+^ less well than dysferlin FerA. The binding of myoferlin FerA to PS-containing vesicles is significantly more robust in the presence of Ca^2+^ (Fig. 6B). Surprisingly, otoferlin FerA is able to bind PC-only vesicles modestly well with or without Ca^2+^; however, in the presence of negatively-charged phospholipids, otoferlin FerA becomes Ca^2+^-independent, and it quantitatively binds to liposomes regardless of vesicle composition. In total, these results are consistent with dysferlin FerA binding to phospholipid surfaces in the presence of Ca^2+^.

## Discussion

The C2A domain of dysferlin has been shown to be thermodynamically weak (ΔG of unfolding <1 kcal/mol), a property thought to be advantageous in interacting with dynamic phospholipid membranes^5,39^. Since other C2 domains, from other proteins, are also moderately unstable^39^, it is likely that all the C2 domains in the ferlins are similarly fragile. Interestingly, the stability of the FerA domain is on the same order as that of dysferlin C2A (ΔG of unfolding of 1.47 kcal/mol at 37 °C)^5^. The isolated FerA domain of dysferlin, myoferlin, and otoferlin can unfold and refold multiple times without obvious signs of deterioration (Fig. S5), a property not shared by the C2 domains of dysferlin^5^. It is clear that FerA is a unique structural domain within the ferlin proteins, but the function of the FerA domain within the ferlin proteins is still unknown.

To probe the function of the FerA domain, we studied the structural and thermodynamic properties of two clinically-defined mutations of dysferlin FerA, V705M, and P731R. Muscular-dystrophy patients carrying the V705M dysferlin variant have been diagnosed with Miyoshi Myopathy^40^, and present with weakness in the distal skeletal muscles. Analysis of V705M by DSC and CD revealed that the stability of the domain had been compromised (Table 1 and Table S2). The resulting ΔG was measured to be 0 kcal/mol, hence 50% of the domain can be expected to be unfolded at 37 °C. Further, there is an increased ANS accessibility, confirming increased exposure of the hydrophobic core relative to that of wild-type FerA (Fig. 5). Yet, there is no measurable decrease in the fraction of secondary structure due to this mutation (Fig. S3 (inset), Table S3). These data strongly suggest helical bundle reorganization to accommodate the larger methionine residue. Indeed, the four-helix bundle ColE1 Rop experienced profound reorganization with a similar mutation^41^.

The P731R mutation in dysferlin results in a similar clinical diagnosis to Miyoshi Myopathy, that of Limb-Girdle Muscular Dystrophy-Type 2B. Patients are reported to be “still ambulant”, but with plasma creatine-kinase levels increased ca. 10-fold^40,42^. As with the V705M mutation, the secondary structure of the P731R FerA domain is not affected (Fig. S3 (inset), Table S3), but the stability of the domain is compromised (Table 1, Fig. S3, Table S2), albeit not to the same extent as with V705M. Secondary-structure prediction of FerA indicates that Pro-731 occurs on the long B-C linker (Fig. 1). A reasonable conclusion would be that a proline substitution may compromise a hinge region in the linker that may be necessary for domain mobility. Pro-731 is conserved only between dysferlin and Fer1L5, which argues against this residue as a structural element essential for a hinging motion in FerA. However the other ferlin paralogs, with the exception of otoferlin, possess proline residues within the B-C linker. It is possible that the replacement of a conformationally restrictive residue such as proline with a more flexible residue such as arginine may allow for more freedom of motion between helices A-B and C-D.

To test if FerA domains bind to membrane similar to their surrounding C2 domains, the membrane-binding activity of FerA was monitored using a cosedimentation assay. Surprisingly, we discovered Ca^2+^-dependent binding for the FerA domains of dysferlin and myoferlin; however, unlike the C2 domains that flank the FerA domain, they do not appear to be specific for negatively-charged phospholipids (Fig. 6). In the absence of Ca^2+^, dysferlin FerA shows a marginal propensity to bind uncharged lipid (100% Phosphatidylcholine, PC); but, in the presence of Ca^2+^, the binding of FerA to liposomes increases markedly. The V705M mutation has similar liposome-binding activity without Ca^2+^, but it binds more robustly in the presence of Ca^2+^. It is possible that the less thermodynamically stable V705M mutant unfolds more easily in the presence of Ca^2+^ and membrane. The unique ability of V705M to unfold due to its core packing mutation is consistent with our observations from the Gibbs-Helmholtz plot (Fig. 4) and its augmented ANS accessibility (Fig. 5). The relative ease of unfolding and increased propensity to associate with phospholipid could explain the pathogenicity of this mutation. The liposome binding activity of P731R is similar to wild-type without Ca^2+^. There is a clear decrease in the response of P731R to 100% PC liposomes in the presence of Ca^2+^ compared to wild-type and V705M measured under the same conditions. However, there is no significant difference between wild-type dysferlin FerA and P731R FerA in the presence of negatively-charged phospholipid; therefore, the pathogenicity of P731R may be related to its loss of thermodynamic stability.

Next, we studied the liposome-binding activity of myoferlin and otoferlin FerA to shed light on the phospholipid-binding diversity among the various ferlin family members. In this assay, myoferlin FerA prefers negatively-charged phospholipids over neutral phospholipids in the presence of Ca^2+^, in contrast to dysferlin FerA. Myoferlin FerA in the absence of Ca^2+^ shows relatively low levels of liposome binding with either 100% Phosphatidylcholine- (PC) or Phosphatidylcholine/Phosphatidylserine-containing (PC/PS) liposomes; however, in the presence of Ca^2+^, binding of PC/PS liposomes increases markedly (Fig. 6). The specificity of myoferlin FerA for negatively-charged phospholipids and Ca^2+^ could reflect the unique phospholipid distribution that is present in myotubes, where myoferlin is most highly expressed^20^, during myoblast formation^43^. Indeed, myoblasts deprived of Ca^2+^ do not fuse into myotubes^44^. The membrane binding results with otoferlin FerA were unique. In the absence of Ca^2+^, almost 25% of the otoferlin FerA bound to PC membranes (Fig. 6). That fraction increased two-fold in the presence of Ca^2+^. However, in the presence of negatively-charged liposomes, otoferlin FerA became Ca^2+^-independent and otoferlin FerA quantitatively bound to liposomes. In this case, there could be a substantial electrostatic attraction between the FerA domain and the liposome surface prior to any membrane-binding events.

Our data shows that the phospholipid interaction of FerA domains is enhanced by the presence of Ca^2+^. Indeed soluble FerA does not bind Ca^2+^ as measured by ITC (data not shown). As there are no consensus Ca^2+^ binding sites on the surface of the ferlin domain, a question remains as to where Ca^2+^ binds or how Ca^2+^ enhances FerA’s lipid binding activity. One possibility is that FerA transitions from a soluble four-helix bundle to an inverted membrane-associating domain with the hydrophobic core directed toward the hydrophobic portion of the bilayer similar to the transitions observed in colicin^45^. The hydrophilic residues would be directed toward the center of the helical bundle. Acidic residues from neighboring helices could form a nascent Ca^2+^ binding site that could stabilize the bundle in the presence of membrane. An up-down-up-down four-helix bundle topology could position these residues in close proximity to form a Ca^2+^ binding site upon inversion near a membrane. Indeed, there are conserved aspartic and glutamic acid residues on Helices A and D of dysferlin and myoferlin (Fig. 1), but not in otoferlin.

In conclusion, we have shown that the FerA domain of the ferlin proteins is a four-helix bundle unique to the ferlin family of proteins. Each FerA domain possesses its own net charge that is likely important for its overall function in the cell type and membrane environment in which it functions. Given the membrane fusion role that ferlins contribute to the cell, there are two possible roles that FerA could serve in the ferlin molecule. First, FerA could play a SNARE-like role, where the zippering of helices A-B with C-D are similar to the zippering of the v-SNAREs helices with t-SNARE helices^46^. Therefore, in this model, ferlin proteins could be thought of as a composite of synaptotagmin and SNAREs. The Ca^2+^-sensing activity would be contributed by the C2 domains of the ferlins, and the fusogenic SNARE activity would be mediated by the FerA four-helix bundle. Indeed, almost all of our computed models of dysferlin FerA have a single Arg residue (Arg-744, Helix C) at the center of the four-helix bundle similar to the SNARE bundle^10^. This arginine residue is also conserved in myoferlin FerA (Arg-726), but not in otoferlin FerA. A second, more likely possibility, is that the FerA domain could act like a virus fusion peptide^47^. Its insertion into the membrane would induce negative curvature thus catalyzing membrane fusion between the patching vesicle and the target membrane^48^. Further experiments will be needed to test these hypotheses.

## Methods

### Cloning of FerA Genes

DNA encoding the relevant FerA domain sub-sequences were extracted from human dysferlin (residues 670–783) [GenBank AAC63519.1], human myoferlin (residues 610–723) [GenBank AAF27177.1], and human otoferlin (residues 723–839) [Swiss-Prot Q9HC10.3] and were synthesized by GeneWiz as codon-optimized genes for *E. coli* expression. The codon-optimized, synthetic FerA genes were sub-cloned into a pET28A-MBP (maltose binding protein) expression plasmid with a TEV protease cleavage site located between the FerA gene and His-tagged MBP. Recombinant plasmids were isolated from corresponding transformed colonies. The nucleotide sequences of the resulting recombinant expression plasmids were confirmed by DNA sequencing from GeneWiz. Point mutations corresponding to V705M and P731R were introduced into the wild-type dysferlin FerA constructs using the Quikchange site-directed mutagenesis protocol, and confirmed with DNA sequencing by GeneWiz.

### Expression and Purification of FerA

Expression plasmids containing dysferlin FerA, dysferlin FerA mutants, myoferlin FerA, and otoferlin FerA were transformed into chemically competent BL21(DE3) cells, spread onto fresh kanamycin plates and incubated overnight at 37 °C. Colonies were picked and used to start a 10-mL overnight inoculum in Luria Broth (LB) plus 50 *μ*g/mL kanamycin at 37 °C. The overnight culture was used to inoculate a 1 L flask of TB (Terrific Broth) plus 50 μg/mL kanamycin at 37 °C. Once the culture reached an OD600 of 2.0, the culture was cooled to 18 °C, then 400 μL of 1 M IPTG was added and the culture was allowed to grow for 12 hours at 18 °C while shaking at 250 rpm in a baffled Fernbach flask. The cells were collected and flash frozen in liquid nitrogen until ready for use. The cells were thawed in lysis buffer (20 mM HEPES, pH 7.4, 200 mM NaCl, 1 mM CaCl_2_) and ruptured with a Microfluidizer. Lysed cells were spun using Beckman JA-20 rotor at 19,500 rpm (45,900 × g) for 45 min. Clarified supernatant was passed through a Ni-NTA affinity column. The resin was washed with lysis buffer until the OD280 was <0.01. The column was then washed with lysis buffer plus 30 mM Imidazole. H(6)-MBP-FerA was eluted with lysis buffer plus 300 mM imidazole. The fusion protein was cleaved with TEV protease overnight at 4 °C. In the cases of the dysferlin and myoferlin FerA domains, each was buffer exchanged into 20 mM HEPES, pH 7.4, 50 mM NaCl and loaded onto a QAE-Sepharose column. The dysferlin FerA domain was eluted using a gradient from 0 to 1 M NaCl, while myoferlin FerA was collected from the flow-through fraction. The fractions containing the FerA domains were collected and the remaining contaminants were removed using a Superdex 200 column; however, otoferlin FerA was selected for on a SP-Sepharose column with the same gradient. Purity was assessed using SDS PAGE Stain-Free gels from BioRad. Stain-Free gels require tryptophan to form the fluorophore necessary for imaging. Otoferlin FerA lacks tryptophan, so Coomassie was used in that case (Fig. S1). Myoferlin and dysferlin FerA domains were quantitated by OD280 using the their calculated extinction coefficients. Otoferlin FerA was quantitated using the BioRad dye-binding assay. The total yield for each purified domain was between 8–12 mg/L of bacterial culture.

### Circular Dichroism

Purified FerA protein samples were dialyzed into 25 mM phosphate buffer, pH 7.4. Circular dichroism was performed on a J-850 spectropolarimeter from JASCO Corp. Prior to analysis, each dialysis protein sample was subjected to SDS-PAGE under reducing conditions to estimate relative protein concentration following Coomassie staining. By comparison to BCA protein assay, all samples demonstrated equivalent protein concentration of 1.3 mg/mL (data not shown) allowing comparison of CD data obtained for each dialysis step sample. Circular dichroism spectra were acquired at 22 °C in the 190–260 nm spectral range at an acquisition rate of 1 nm/sec and a data pitch of 0.1 nm. Three CD spectra of each sample were averaged to calculate the final CD data. CD spectra were also measured for each dialysis buffer and subtracted from the respective protein containing sample spectra. The resulting spectra of all FerA samples were normalized to mean residue ellipticity and deconvoluted using the CONTIN option in DichroWeb to obtain the secondary structure fraction of the samples^49^.

### Secondary-Structure Prediction

Secondary-structure predictions were computed using www.PredictProtein.org^50^.

### Modeling of FerA domain

A BLAST search of the PDB data bank using FerA sequences does not reveal any homologous structures; however, 2QUP “Crystal structure of uncharacterized protein BH1478 from *Bacillus halodurans*”^32^ is 15.2% identical and 25% similar over the entire length of the dysferlin FerA sequence. The alignment of FerA with 2QUP was used in Modeller^30^ to build and refine a homology model for subsequent SAXS analysis (Fig. S4). Robetta was also used to generate sets of likely models^31^. The set of Robetta models and the 2QUP-based model was scored against the SAXS data to access overall quality of the fit.

### Small Angle X-ray Scattering

All SAXS data were collected using a Rigaku BioSAXS-1000 camera on a FR-E^++^ Cu X-ray source (Rigaku Americas Corporation, The Woodlands, TX). The FerA sample was buffer-exchanged and concentrated in a buffer of 100 mM NaCl, and 10 mM sodium phosphate pH 7 using a well-washed Centricon concentrator. The concentrator flow-though was used as the matching buffer and for sample dilution. For each FerA sample a dilution series of 5, 2.5, and 1.2 mg/mL was run, each with a matching buffer. Each SAXS sample used 30 μL of sample manually pipetted into a thin-walled quartz capillary cell, sealed, and mounted in the BioSAXS camera under vacuum. For each sample a series of twelve 30-minute exposures was collected plus a matching buffer. Data were collected in the range 0.008 Å^−1^ < q < 0.68 Å^−1^, and analysis used all significant data to 0.50 Å^−1^. Buffer subtraction, absorption correction, and MW calibration were performed using the SAXNS-ES server (http://xray.utmb.edu/SAXNS), which also uses the concentration- and intensity-independent method of Rambo and Tainer^51^ to determine the molecular weight. Data analysis, including merging of curves, was performed with the Primus program and the P(r) was calculated using DATGNOM, both from the ATSAS suite^52^. The *ab initio* molecular shape was generated from an average of 15 DAMMIF runs^53^, using the SAXNS DAMMIF utility. The GASBOR *ab initio* molecular shape was generated from a cluster analysis of five GASBOR runs^54^, using the SAXNS GASBOR utility. Missing residues were added to the homology models using CORAL. CORAL models were re-scored using CRYSOL to include the hydration-layer contribution.

### Differential Scanning Calorimetry

Differential scanning calorimetry (DSC) analysis was performed using a TA-instruments nano-DSC calorimeter to investigate possible folding differences between wild-type and mutant FerA. The capillary sample cell was loaded with 700 *μ*L of protein at 30 *μ*M. The reference cell was loaded with dialysis buffers. Buffers contained 20 mM MOPS, 100 mM KCl, pH 7.4 (Fig. S5). Samples and buffers were degassed for 15 min under vacuum with magnetic stirring prior to running calorimetric experiments. The heating protocol was executed at a constant rate of 2 °C/min in a 20–100 °C temperature range after 15 min of equilibration time. Data were analyzed with NanoAnalyze software from TA-instruments and the sigmoid method was used for background subtraction.

### ANS Fluorescence

Dysferlin FerA wild-type and mutants were buffer-exchanged into 20 mM HEPES, 300 mM NaCl, pH 7.4. 100 *μ*L of each sample at a concentration of 0.1 mg/mL was incubated with 100 *μ*M 8-anilino-1-naphthalenesulfonic acid (ANS), AnaSpec. Fluorescence of protein-bound ANS and ANS in buffer solution was recorded at 25 °C in a 96 well plate using a BioTek Synergy4 Multimode Plate Reader. ANS was excited at 365 nm and the fluorescence was recorded from 400 to 600 nm with excitation/emission slit width of 5 nm. Data were averaged from three independent measurements.

### Co-sedimentation Assay

Protein-free unilamellar liposomes containing 100% POPC or 60% POPC/40% POPS (Avanti Polar Lipids) were prepared by extrusion through 100 nm polycarbonate filters using an Avanti Mini Extruder. The hydrodynamic diameter of the extruded liposomes was confirmed to be 100 +/− 12 nm, as determined using a Malvern Zetasizer Nano ZS (data not shown). For the co-sedimentation assay, protein samples were buffer-exchanged into Chelex-treated 20 mM HEPES, 150 mM NaCl, pH 7.4. This buffer was used to prepare 100 *μ*L of samples containing 1 mM liposomes, 5 *μ*M protein, and either 0.1 mM EGTA or 1 mM CaCl_2_. Samples were incubated and mixed at 37 °C in a shaker incubator set at 100 rpm. Samples were then centrifuged at 65,000 rpm (183,000 × g) using a Beckman TLA-100 rotor for 45 minutes in a Beckman Optima MAX-E table-top ultracentrifuge and the supernatants and pellets were separated. Equal fractions of the supernatants were subjected to SDS-PAGE and proteins were imaged and quantified using a BioRad Criterion Stain Free Imaging System Gel Imager and Image Lab software (Fig. S2). Each co-sedimentation experiment was performed at least three times. In the FerA co-sedimentation experiments, the same amount of protein used for preparation of liposome-protein samples was loaded as the input control. We also loaded two more samples containing the same amount of protein in addition to either Ca^2+^ or EGTA (no phospholipid) to confirm that FerA does not aggregate in the presence of Ca^2+^ or EGTA (Fig. S2). All these samples including the input control and no-phospholipid controls were treated the same. After loading the samples on SDS-PAGE and quantification of bands, all bands were normalized relative to the input control. This ratio represents the unbound fraction of proteins in the presence of phospholipids. For ease of representation, the data were converted to fraction of protein bound by subtracting the value of unbound from 1; therefore, Fig. 6 represents the fraction of FerA bound to vesicles^55^.

## Electronic supplementary material

Supplemental Information
