# Supplementary material for: FerA is a Membrane-Associating Four-Helix Bundle Domain in the Ferlin Family of Membrane-Fusion Proteins
Source: Sci Rep. 2018 Jul 19;8:10949. doi: 10.1038/s41598-018-29184-1 (PMC6053371; doi:10.1038/s41598-018-29184-1)
Supplement: Supplementary file 1 — Supplemental Information [file 41598_2018_29184_MOESM1_ESM.pdf]

Manuscript submitted to Nature Scientific Reports
Article

SI:FerA is a membrane-associating four-helix bundle domain in the Ferlin family of membrane-fusion proteins

Faraz M. Harsini[1], Sukanya Chebrolu[1], Kerry L. Fuson[1], Mark A. White[2], Anne M. Rice[3], and R. Bryan Sutton[1,4,*]

[1]Texas Tech University Health Sciences Center, Department of Cell Physiology and Molecular Biophysics, Lubbock, TX, 79430-6551, USA
[2]The University of Texas Medical Branch, Department of Biochemistry and Molecular Biology, Galveston, TX, 77555, USA
[3]Johns Hopkins University, Department of Biophysics, Baltimore, MD, 21205, USA
[4]Texas Tech University Health Sciences Center, Center for Membrane Protein Research, Lubbock, TX, 79430-6551, USA
[*]Correspondence: roger.b.sutton@ttuhsc.edu

ABSTRACT Ferlin proteins participate in such diverse biological events as vesicle fusion in *C. elegans*, fusion of myoblast membranes to form myotubes, Ca^{2+}-sensing during exocytosis in the hair cells of the inner ear, and Ca^{2+}-dependent membrane repair in skeletal muscle cells. Ferlins are Ca^{2+}-dependent, phospholipid-binding, multi-C2 domain-containing proteins with a single transmembrane helix that spans a vesicle membrane. The overall domain composition of the ferlins resembles the proteins involved in exocytosis; therefore, it is thought that they participate in membrane fusion at some level. But if ferlins do fuse membranes, then they are distinct from other known fusion proteins. Here we show that the central FerA domain from dysferlin, myoferlin, and otoferlin is a novel four-helix bundle fold with its own Ca^{2+}-dependent phospholipid-binding activity. Small-angle X-ray scattering (SAXS), spectroscopic, and thermodynamic analysis of the dysferlin, myoferlin, and otoferlin FerA domains, in addition to clinically-defined dysferlin FerA mutations, suggests that the FerA domain interacts with the membrane and that this interaction is enhanced by the presence of Ca^{2+}.

SUPPLEMENTAL INFORMATION (SI)

Cloning and Expression of FerA domains

All FerA genes were synthesized by GeneWiz, and supplied in pUC57 vectors for subcloning. All constructs were subsequently subcloned into the p202 expression vector using the Nde1/Xho1 restriction sites. p202 is based on the pET28A backbone and integrates a His_6-tagged Maltose Binding Protein (MBP) fusion protein in-frame with the pET28A multi-cloning site. The His_6-tagged MBP can be removed by incubation with recombinant Tobacco Etch Virus Protease (TEV). All of the FerA expression vectors were grown in *E. coli* BL21(DE3) in 1L of TB media at 37 °C to an OD_{600}=2 and cooled to 18 °C. Heterologous expression of His_6-MBP-FerA was induced with 400 μM IPTG. Expression continued at 18 °C for 18 hours in baffled Fernbach flasks at 250 rpm. Cells were harvested by centrifugation, flash frozen and kept in liquid nitrogen until needed.

Purification of Wild-type Dysferlin FerA, V705M, and P731R

Fifteen grams of BL21(DE3) were lysed in lysis buffer (150 mM NaCl, 20 mM HEPES, 5 mM $CaCl_2$, pH 7.4) using a Microfluidics M110-EH30 Microfluidizer Processor. The cell lysate was centrifuged in a JA-20 rotor at 19,500 rpm (45,900 x g), at 4 °C for 45 min. After the spin the supernatant was passed over a Ni^{2+}-NTA affinity column (Thermo-Fisher). The column was washed with lysis buffer until the OD_{280} was <0.01. The resin was washed again in wash buffer (150 mM NaCl, 20 mM HEPES, 5 mM $CaCl_2$, 30 mM Imidazole, pH 7.4). $CaCl_2$ was included in all wash buffers to maintain consistency with the C2 domain purification protocols. The His_6-MBP-FerA fusion protein was then eluted from the column using 50 ml of a solution containing 150 mM NaCl, 20 mM HEPES, 5 mM $CaCl_2$, 300 mM Imidazole (pH 7.4). The His_6-MBP-FerA was then cleaved with TEV protease at 4 °C for 12 hours. The cleaved protein was buffer-exchanged into Buffer A (20 mM HEPES, 50 mM NaCl, and 5 mM $CaCl_2$, pH 7.4) to prepare for IEX (QAE-Ion Exchange Chromatography). The protein was injected onto a QAE-Sepharose protein and then washed at a conductivity <26 mS/cm. The dysferlin FerA domain elutes from the QAE-Sepharose resin as a single peak starting at approximately 26 ms/cm using a 0 to 1 M NaCl gradient. The tubes containing the peak were collected and further purified using an 80-cm Superdex 75 gel filtration column. The buffer used for gel filtration is 150 mM NaCl, 20 mM HEPES, 5 mM $CaCl_2$ (pH 7.4) (Fig. S1).

Purification of Wild-type Myoferlin FerA

Fifteen grams of BL21(DE3) were lysed in lysis buffer: 150 mM NaCl, 20 mM HEPES, 5 mM $CaCl_2$ (pH 7.4) using a Microfluidics M110-EH30 Microfluidizer Processor. The cell lysate was centrifuged in JA-20 rotor at 4 °C for 45 min at 19,500 rpm (45,900 x g). After the spin the supernatant was passed over a Ni^{2+}-NTA affinity column (Thermo-Fisher). The column was washed with lysis buffer until the OD_{280} was <0.01. The resin was washed again in wash buffer: 150 mM NaCl, 20 mM HEPES, 5 mM $CaCl_2$, 30 mM Imidazole (pH 7.4). The His_6-MBP-FerA fusion protein was then eluted from the column using 50 ml of 150 mM NaCl, 20 mM HEPES, 5 mM $CaCl_2$, 300 mM Imidazole (pH 7.4). The His_6-MBP-FerA was then cleaved with TEV protease at 4 °C for 12 hours. The cleaved protein was buffer exchanged into Buffer A: 20 mM HEPES, 50 mM NaCl, and 5 mM $CaCl_2$, (pH 7.4) to prepare for IEX (QAE - Ion Exchange Chromatography). The protein was injected onto a QAE-Sepharose protein and then washed in Buffer A. The myoferlin FerA does not bind to the column and elutes with the flow through material. The flow through peaks were collected, concentrated, and applied to an 80 cm Superdex 75 Gel filtration column. The buffer used for Gel filtration is 150 mM NaCl, 20 mM HEPES, 5 mM $CaCl_2$, 5 mM Maltose, and one AEBSF tablet to prevent proteolysis (pH 7.4) (Fig. S1).

Ferlin FerA Domain	Residue Range	Net Charge at pH 7.0	calculated pI
Dysferlin	844-963	-8.4	5.03
Myoferlin	652-765	-1.6	5.97
Otoferlin	723-839	+8.5	9.51
Fer1L4	775-890	+20.3	11.65
Fer1L5	583-689	+9.1	9.77
Fer1L6	615-732	-0.6	6.58

Table S1: Estimated Net charge of FerA domains from the six known ferlin proteins from humans. Dysferlin; Myoferlin; Otoferlin; Fer1L4 [GenBank: AAS19932.1]; Fer1L5 [RefSeq: XP_011510412.1]; Fer1L6 [RefSeq: NP_001034201.2].

Purification of wild-type Otoferlin FerA

Fifteen grams of BL21(DE3) were lysed in lysis buffer: 150 mM NaCl, 20 mM HEPES, 5 mM $CaCl_2$ (pH 7.4) using a Microfluidics M110-EH30 Microfluidizer Processor. The cell lysate was centrifuged in JA-20 rotor at 4 °C for 45 min at 19,500 rpm (45,900 x g). After the spin the supernatant was passed over a Ni^{2+}-NTA affinity column (Thermo-Fisher). The column was washed with lysis buffer until the OD_{280} was <0.01. The resin was washed again in wash buffer: 150 mM NaCl, 20 mM HEPES, 5 mM $CaCl_2$, 30 mM imidazole (pH 7.4). The His_6-MBP-FerA fusion protein was then eluted from the column using 50 ml of 150 mM NaCl, 20 mM HEPES, 5 mM $CaCl_2$, 300 mM imidazole (pH 7.4), 1 mM DTT. The His_6-MBP-FerA was then cleaved with TEV protease at 4 °Cfor 12 hours. The cleaved protein was dialyzed in a dialysis bag against 20 mM MES, 200 mM NaCl, 5 mM β-mercaptoethanol, 1 mM EDTA at pH 6 at room temperature overnight. After dialysis the protein was spun in a JA-20 rotor at 4 °C for 45 min at 19,500 rpm (45,900 x g). The supernatant was collected and filtered using a 0.45 μm syringe filter. The protein was applied to a SP-Sepharose column and washed with Buffer A. Otoferlin FerA has no tryptophan residues, so it cannot be detected well at OD_{280} or by stain-free gel. The elution peak from otoferlin FerA is weak, due to residual phenylalanine and tyrosine absorbance at OD_{280}. The protein peak can be confirmed by SDS gel with Coomassie staining. Otoferlin FerA typically begins to elute from the SP Sepharose column in a salt gradient beginning at >55 mS/cm under the conditions reported (Fig. S1).

Circular-Dichroism melt

Temperature vs wavelength scan was performed using Variable Temperature Module that allows CD measurements at any given wavelength(s) at a range of temperatures (Fig. S3). In this study, we collected spectra at 222 nm while the temperature was increased from 5 to 70 °C with a bandwidth of 1 nm and Data Integration Time (D.I.T.) of 1 s. Samples were equilibrated at the target temperature for 5 seconds before the measurement was recorded.

Calculated charge on FerA domains

Net charge calculations for each FerA domain were made using `http://protcalc.sourceforge.net/` (Table S1).

Domain	T_M (°C)	ΔH_{vH} (kcal/mol)	TΔS (kcal/mol)	ΔG (kcal/mol)
**Dysferlin FerA WT**	47.4 ±0.18	47.27 ±2.98	45.715 ±2.30	1.53 ±0.066
**Dysferlin FerA V705M**	37.7 ±0.23	31.85 ±1.07	32.871 ±1.398	0.074 ±0.027
**Dysferlin FerA P731R**	43.1 ±0.61	38.90 ±3.00	36.770 ±1.575	0.751 ±0.118

Table S2: Thermodynamic summary of the Dysferlin FerA domain and clinically-derived mutations obtained from Circular-Dichroism (CD) analysis. The average and standard deviation for each measurement is reported. n = 4 for each measurement. T_M was determined from analysis of the CD melts curves by monitoring 222 nm vs temperature. ΔH_{vH} is the van 't Hoff enthalpy calculated from the CD melt data. TΔS is the entropy value computed from the CD melt data. ΔG was calculated at 37 °C .

FerA Domain	α-helix$_R$	α-helix$_D$	β-strand$_R$	β-strand$_D$	turn	unordered	$\text{nRMSD}_{\text{model}}$
**Dysferlin WT**	67.1%	26.6%	0	6.2%	0	0	0.068
**Dysferlin V705M**	67.8%	28.2%	0	1.4%	0	2.5%	0.053
**Dysferlin P731R**	67.3%	25.0%	0	5.1%	0	2.7%	0.059
**Myoferlin**	60.2%	26.0%	0	1.8%	2.1%	9.9%	0.045
**Otoferlin**	66.9%	27.8%	0	1.3%	0.2%	3.8%	0.050

Table S3: Predicted secondary structure content of ferlin FerA domains from analysis of Circular-Dichroism spectra. α-helix$_R$ is defined as regular α-helix. α-helix$_D$ is defined as distorted α-helix. β-strand$_R$ is defined as regular β-strand. β-strand$_D$ is defined as distorted β-strand. nRMSD is defined as normalized root-mean-square deviation of the model spectra versus the experimental spectra.

	**Dysferlin FerA-1**	**Dysferlin FerA-2**	**Dysferlin FerA-3**	**Dysferlin FerA-4**
**Concentration (mg/ml)**	5	2.5	1.2	**merged**
**Exposure (min)**	60	60	30	*
**Q-range (Å^{-1})**	0.018-0.68	0.021-0.68	0.018-0.68	**0.018-0.64**
**R_g(Å)**	16.5±0.3	16.2±0.7	16 ±1	**16.4 ±0.4**
**D_{max}(Å)**	56	53	49	**54**
**Shanum(Å^{-1})**	0.60	0.65	0.58	**0.64**
**MW (kDa)**	14.8	12.2	12.5	**14.6**
**MW_{RAMBO} (kDa)**	14.2	12.8	14.0	**14.0**
**Crysol $\tilde{\chi}^2$ (2QUP)**	2.0	0.9	1.7	**1.5**
**CORAL M1 $\tilde{\chi}^2$**	–	–	–	**1.1**
**CORAL M2 $\tilde{\chi}^2$**	–	–	–	**0.9**
**CORAL M3 $\tilde{\chi}^2$**	–	–	–	**0.9**
**CORAL M4 $\tilde{\chi}^2$**	–	–	–	**0.9**
**CORAL M5 $\tilde{\chi}^2$**	–	–	–	**14**

Table S4: Small Angle X-ray Scattering (SAXS) and model fitting parameters

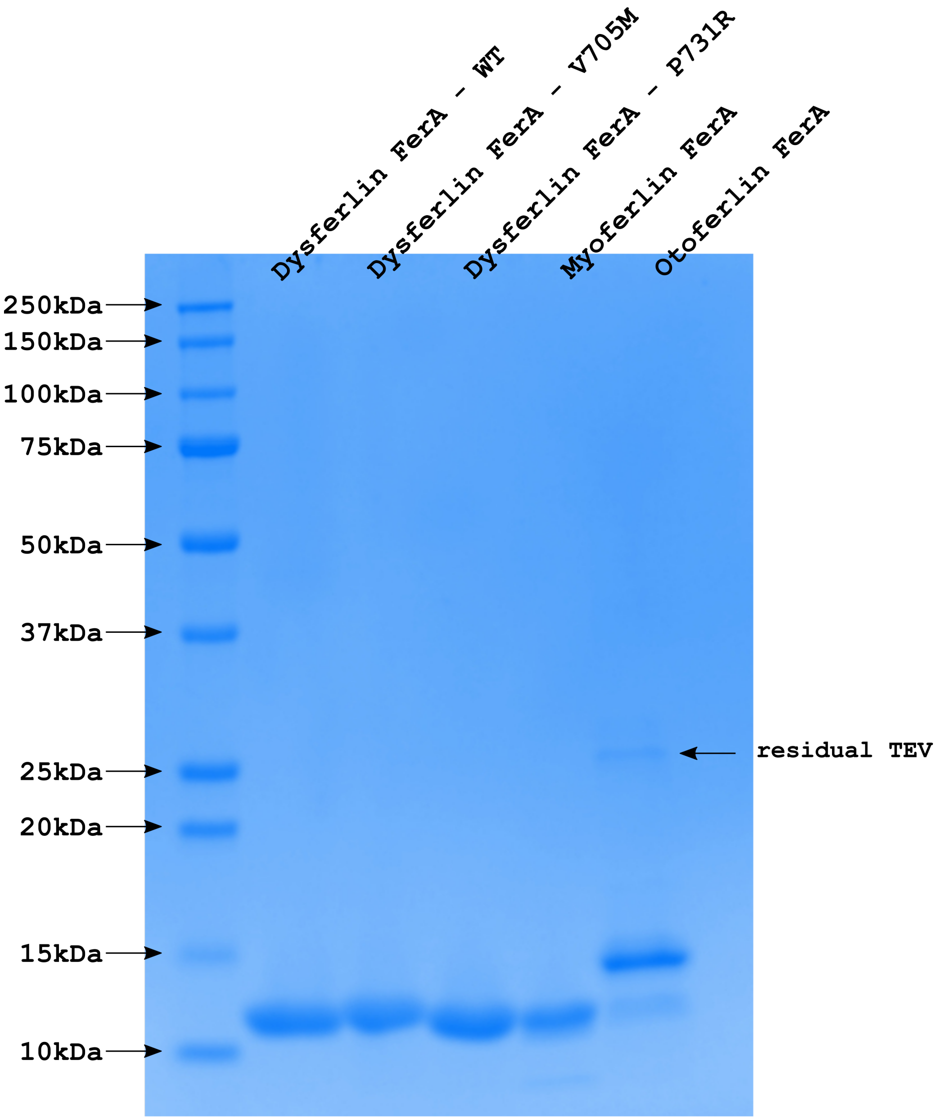

Figure S1: Full-format SDS-PAGE Coomassie-stained gel of FerA domain purification. Molecular weight ladder is in the far-left lane. Dysferlin WT, V705M, P731R, myoferlin, and otoferlin FerA domains are listed from left to right. A minor TEV contamination is noted in the otoferlin FerA preparation. In addition, some proteolytic cleavage that occurred in some preparations in myoferlin FerA and otoferlin FerA. Otoferlin FerA does not possess Trp residues, so this 15-25% PAGE-SDS gel is stained with Coomassie Blue.

A

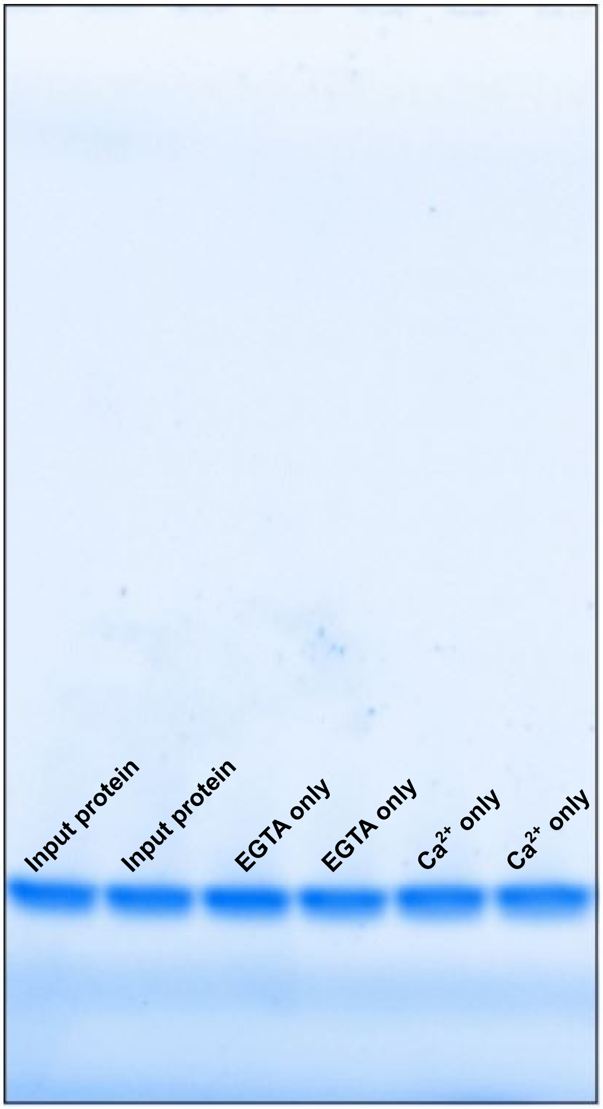

B

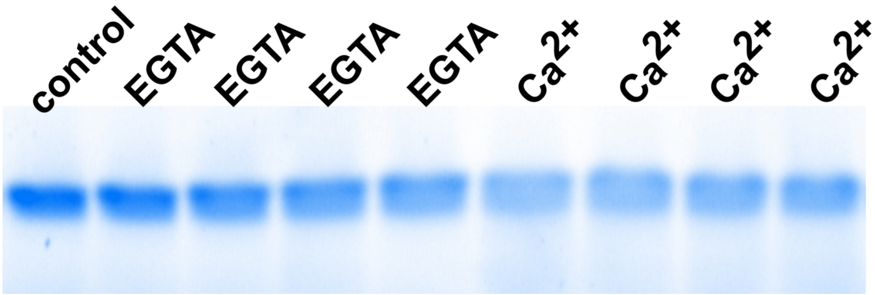

Figure S2: **Example Co-sedimentation Gel.** A. Full-format SDS-PAGE gel of co-sedimentation controls. B. Cropped SDS-PAGE gel of highly purified dysferlin FerA samples used for co-sedimentation. Replicates of four lanes of EGTA + vesicles + FerA; and four lanes of Ca^{2+} + vesicles + FerA. 10 - 15% PAGE Gel stained using BioRad Stain-Free system.

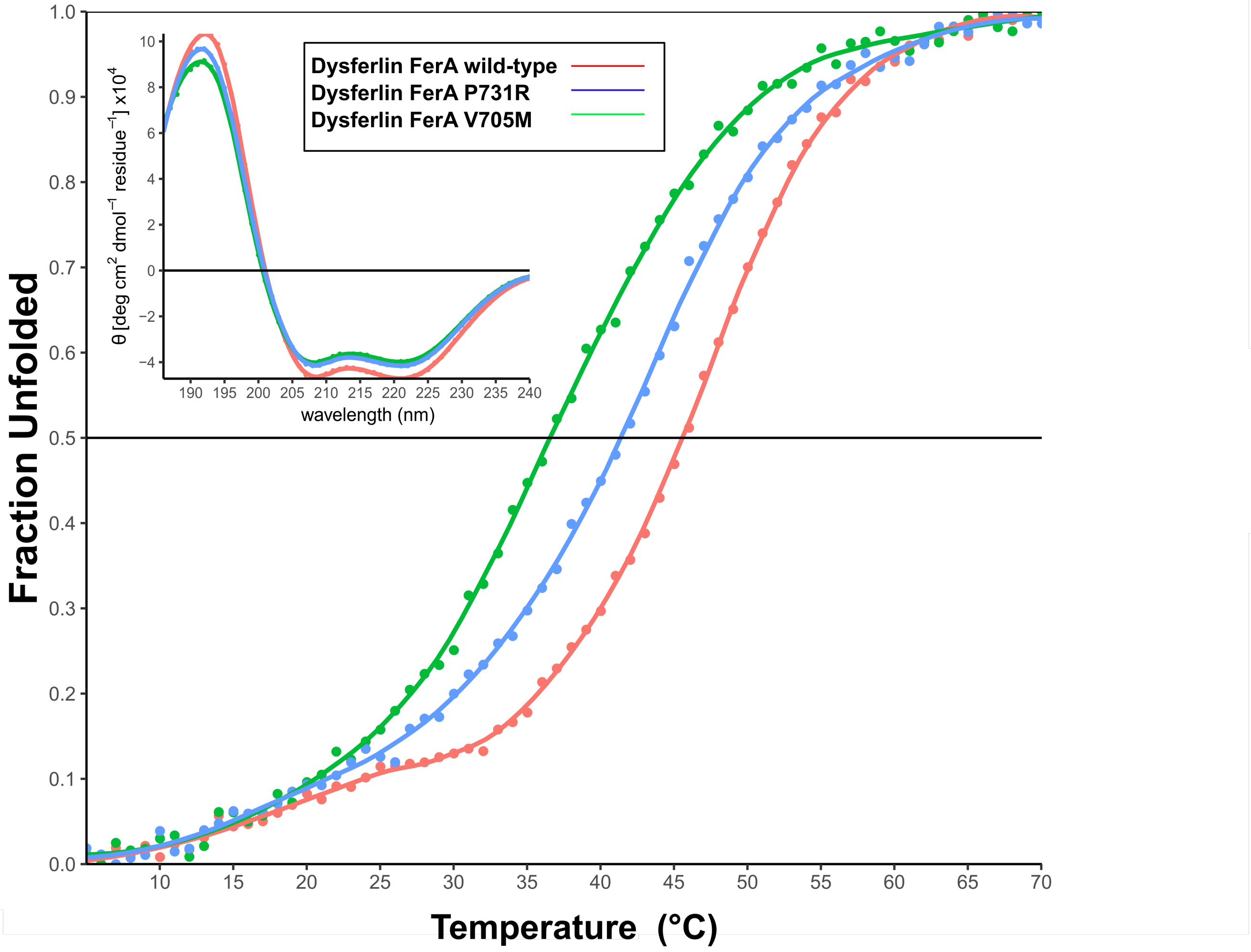

Figure S3: **Circular Dichroism thermal melts of dysferlin mutants** Circular-Dichroism melts of wild-type dysferlin FerA (red), V705M (green), P731R (blue). The spectra were normalized from 0 to 1 (100% unfolded). The molar ellipticity vs. wavelength spectra of each construct is included as an insert in the upper left.

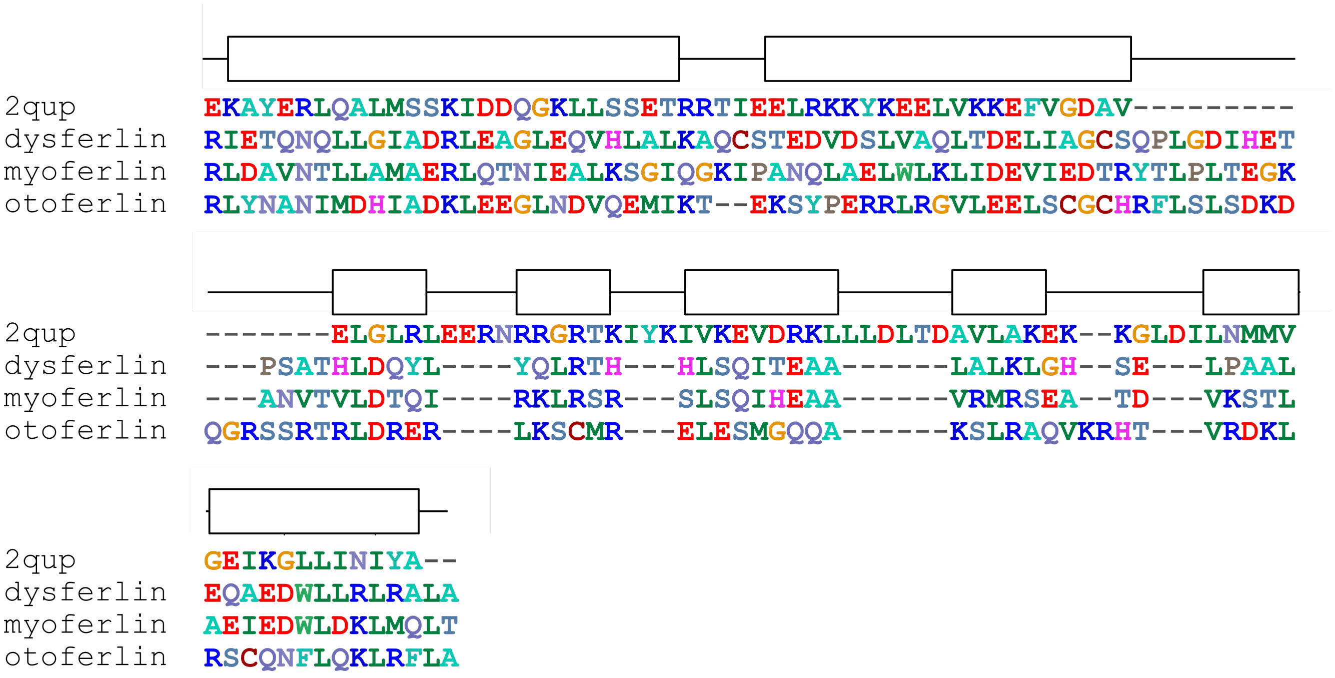

Figure S4: **Primary sequence alignment of dysferlin, myoferlin, and otoferlin FerA with 2QUP.** The alignment was generated using Promals3D. Boxes above the alignment correspond to consensus helices from the 2QUP structure and predicted helices from the FerA domains.

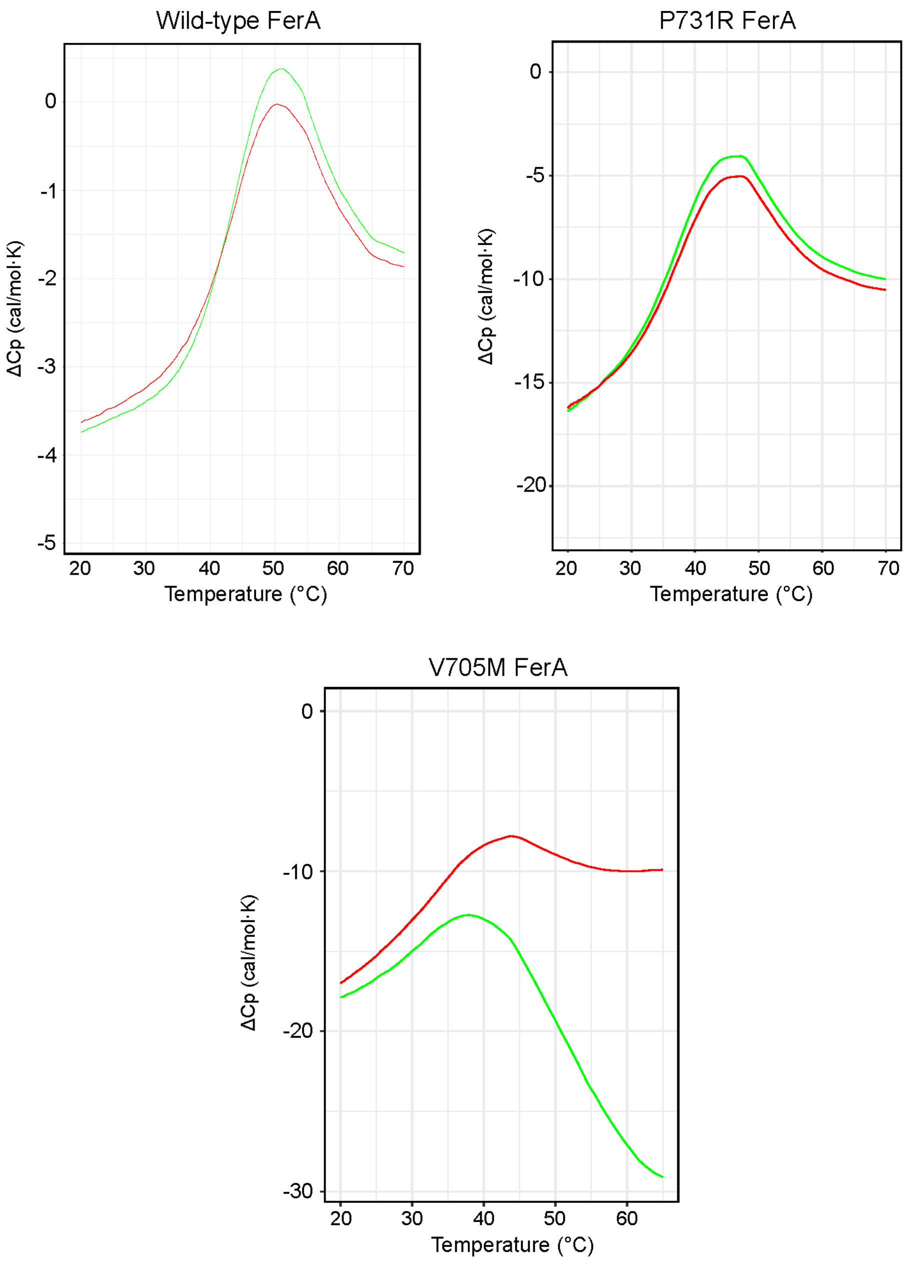

Figure S5: Raw DSC thermogram of wild-type dysferlin FerA domain. Green thermogram, initial melt; red thermogram, refolded melt.

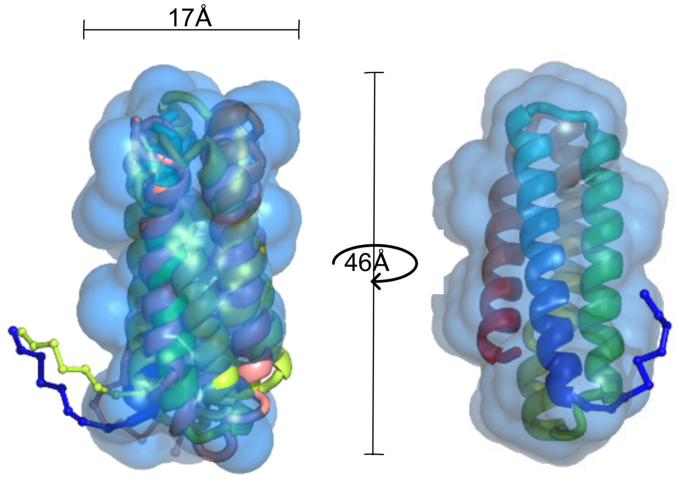

Figure S6: SAXS-constrained FerA *ab initio* and rigid-body models. The superpositioned DAMMIF *ab initio* bead model and the top three Robetta based CORAL models in perpendicular side views. The CORAL model's missing N-terminal residues were modeled as a flexible Cα chain. The Robetta models are colored blue, lime, and salmon.

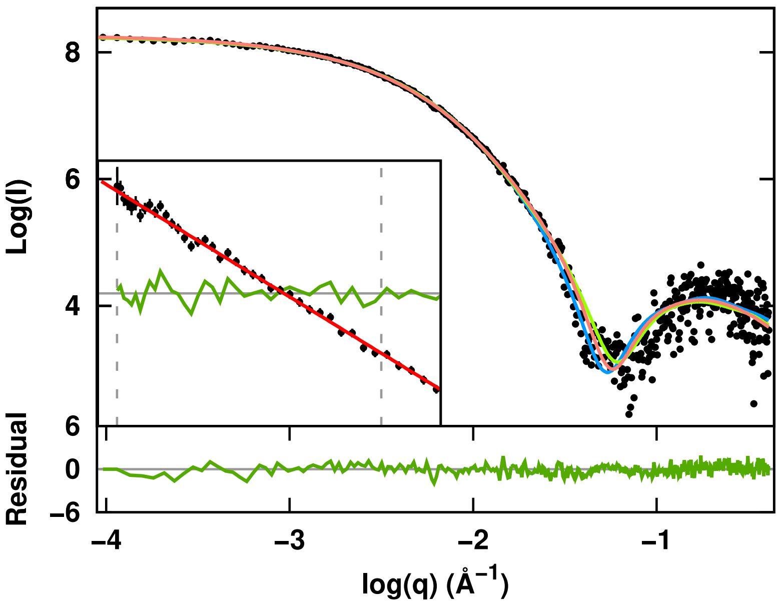

Figure S7: Model fit to SAXS data and Guinier Curve. The FerA SAXS data (•) with the top three CORAL rigid-body model fits (blue, lime, and salmon lines) corresponding to the models in Fig. S6. Bottom curve shows the normalized-residuals of the best fit. Inset: The Guinier fit (red) to the SAXS data (•). The Guinier limits (q_{min}, q 1.3/R_g) are shown as gray vertical dashed lines. The normalized residuals are shown as green lines, with zero residual shown as a gray line.
